# Bacteriophage therapy as an innovative strategy for the treatment of Periprosthetic Joint Infection: a systematic review

**DOI:** 10.1007/s00264-024-06295-1

**Published:** 2024-09-10

**Authors:** Shengdong Yang, Assala Abu Mukh, Elsayed Abdelatif, Axel Schmidt, Cécile Batailler, Tristan Ferry, Sébastien Lustig

**Affiliations:** 1 Department of Orthopedic Surgery and Sport Medicine, FIFA Medical Center of Excellence, Croix-Rousse Hospital, Lyon University Hospital, Lyon, France; 2 IFSTTAR, LBMC UMR_T9406, University Claude Bernard Lyon 1, University of Lyon, Lyon, France; 3 Orthopedics and Traumatology, Vita-Salute San Raffaele University, Milan, Italy; 4 Department of Orthopedic Surgery and Traumatology, Faculty of Medicine, Minia University, Minia, Egypt; 5 Centre interrégional de Référence pour la prise en charge des Infections Ostéo-Articulaires complexes (CRIOAc Lyon), Hospices Civils de Lyon, Lyon, France; 6 Service de Maladies Infectieuses et Tropicales, Hôpital de la Croix-Rousse, Hospices Civils de Lyon, Lyon, France

**Keywords:** Bacteriophage, Periprosthetic joint infection, Hip arthroplasty, Knee arthroplasty

## Abstract

**Background:**

Periprosthetic Joint Infection (PJI) following hip and knee arthroplasty is a catastrophic complication in orthopaedic surgery. It has long been a key focus for orthopaedic surgeons in terms of prevention and management. With the increasing incidence of antibiotic resistance in recent years, finding more targeted treatment methods has become an increasingly urgent issue. Bacteriophage Therapy (BT) has emerged as a promising adjunctive treatment for bone and joint infections in recent years. It not only effectively kills bacteria but also demonstrates significant anti-biofilm activity, garnering substantial clinical interest due to its demonstrated efficacy and relatively low incidence of adverse effects.

**Purpose:**

This review aims to systematically evaluate the efficacy and safety of bacteriophage therapy in treating PJI following hip and knee arthroplasty, providing additional reference for its future clinical application.

**Methods:**

Following predefined inclusion and exclusion criteria, our team conducted a systematic literature search across seven databases (PubMed, Embase, Web of Science, Cochrane Library, ClinicalTrials.gov, CNKI, and WanFang Database). The search was conducted up to May 2024 and included multiple clinical studies on the use of bacteriophage therapy for treating PJI after hip and knee arthroplasty to assess its efficacy and safety.

**Results:**

This systematic review included 16 clinical studies after screening, consisting of 15 case reports and one prospective controlled clinical trial, involving a total of 42 patients with PJI treated with bacteriophage therapy. The average patient age was 62.86 years, and 43 joints were treated, with patients undergoing an average of 5.25 surgeries. The most common pathogen in these infections was *Staphylococcus aureus*, accounting for 18 cases. 33 patients received cocktail therapy, while nine were treated with a single bacteriophage preparation. Additionally, all patients underwent suppressive antibiotic therapy (SAT) postoperatively. All patients were followed up for an average of 13.55 months. There were two cases of recurrence, one of which resulted in amputation one year postoperatively. The remaining patients showed good recovery outcomes. Overall, the results from the included studies indicate that bacteriophage therapy effectively eradicates infectious strains in various cases of PJI, with minimal side effects, demonstrating promising clinical efficacy.

**Conclusion:**

In the treatment of PJI following hip and knee arthroplasty, bacteriophages, whether used alone or in combination as cocktail therapy, have shown therapeutic potential. However, thorough preoperative evaluation is essential, and appropriate bacteriophage types and treatment regimens must be selected based on bacteriological evidence. Future large-scale, randomized controlled, and prospective trials are necessary to validate the efficacy and safety of this therapy.

**Supplementary Information:**

The online version contains supplementary material available at 10.1007/s00264-024-06295-1.

## Introduction

Periprosthetic joint infection (PJI) is a catastrophic complication after joint replacement surgery and has consistently posed a challenging problem for orthopaedic surgeons. Despite advances in surgical techniques and innovative use of antibiotics, the overall incidence of PJI remains at 0.97% for total hip arthroplasty (THA) and 1.03% for total knee arthroplasty (TKA). According to previous surveys, PJI is the most common reason for revision surgery in TKA (25%) and the third most common reason for revision in THA (15.4%) [1, 2]. Further study have compared the impact of different aetiologies on the incidence of PJI following primary joint replacement surgery, revealing that patients with rheumatoid arthritis have a higher incidence compared to those with osteoarthritis [3]. Additionally, PJI is associated with a relatively high mortality and complication rate, which significantly affects patient prognosis. Literature reports a 90-day mortality rate of 0.9% for PJI, with postoperative complication rates of 31.3% for knee joints and 19.6% for hip joints [4], some studies also have reported a five year mortality rate for PJI as high as 26%, comparable to the mortality rates of several common malignancies such as prostate and breast cancer [5]. In terms of healthcare costs, PJI also imposes a substantial financial burden on both patients and society. For example, in the United States, the annual hospital costs related to hip and knee PJI are projected to reach $1.85 billion by 2030 [6].

Currently, once PJI is suspected or diagnosed, various treatment options are available. However, patients almost invariably require additional surgery combined with prolonged antibiotic therapy. Traditional treatment methods are often limited in effectiveness against multidrug-resistant bacteria, and the presence of complex bacterial strains further complicates treatment. Moreover, the side effects of antibiotic therapy add to the patient’s risk. Therefore, there is an urgent need to discover new therapeutic strategies to address these challenges.

Bacteriophages (also known as Phage) are natural viruses that are ubiquitous in the environment and specifically infect and lyse bacteria. They exhibit high specificity and generally do not affect the body’s normal microbiota [7]. Leveraging this advantage, the use of bacteriophages as a method to combat bacteria has gradually emerged as a novel clinical option for bacterial diagnosis and treatment. Bacteriophage therapy has been shown to be effective against infections in various organs and systems, including the pulmonary, urinary, skin wounds, intestinal, and musculoskeletal systems [8–14]. In the field of PJI after joint replacement, from a diagnostic perspective, studies have compared the use of bacteriophage-based detection methods with traditional microbial cultures in sonicate fluid (SF) samples from patients undergoing revision surgery for suspected PJI. Results indicate that bacteriophage-based methods are faster and more sensitive, demonstrating clear advantages [15]. From a therapeutic perspective, a series of foundational studies on bacteriophage treatment for device-related infections have shown promising progress. In in vitro experiments, studies analyzed the bacteriophage activity against *Staphylococcus aureus* isolates from PJI cases, showing that at least one bacteriophage inhibited planktonic bacterial growth in 97% of the samples [16]. In animal studies, research applied phage-coated implants to treat joint infection models in mice. The findings revealed that implants containing bacteriophages were effective in both treating and preventing infections caused by methicillin-resistant *Staphylococcus aureus* (MRSA) strains [17]. Other studies utilized bacteriophage-derived lysins in in vitro models and mouse prosthetic joint infection models, finding that bacteriophage products effectively reduced bacterial presence on peri-prosthetic tissues and implant surfaces [18]. Therefore, bacteriophage therapy shows significant potential in the clinical management of PJI, leveraging its high specificity and low side effects to target specific bacteria effectively and act rapidly against antibiotic-resistant strains.

Currently, a growing number of clinical studies are focusing on the efficacy and mechanisms of bacteriophage therapy in managing PJI. Thus, this review aims to systematically evaluate the efficacy and safety of bacteriophage therapy in treating PJI following hip and knee arthroplasty, providing further insights for its future clinical applications.

## Materials and methods

### Literature search strategy

In May 2024, we conducted a systematic search of the literature on the role of bacteriophages in managing PJI based on the Preferred Reporting Items for Systematic Reviews and Meta-Analyses (PRISMA) guidelines [19]. Specifically, we formulated our search strategy based on the PICO framework, which includes predefined parameters for Population, Intervention, Comparison, Outcome, and Study Design. The study population consisted of patients with PJIs following hip or knee arthroplasty. The intervention involved the use of bacteriophages (or derived biological agents like lysins) administered through various routes. The comparison, where applicable, was against traditional standard treatment regimens such as antibiotic therapy alone. The primary outcomes were infection clearance rates and clinical recovery. The search was conducted by two authors who screened seven databases (PubMed, Embase, Web of Science, Cochrane Library, Clinical Trials, CNKI, and WanFang Database). Various search terms were employed, including “hip arthroplasty,” “knee arthroplasty,” “bacteriophage therapy,” and “postoperative infection” (for detailed search strategies, see the appendix). Additionally, we manually searched the reference lists of relevant articles to ensure all pertinent studies were included. The search covered the period from the inception of the databases to the present.

### Inclusion and exclusion criteria

Inclusion criteria: Clinical studies involving bacteriophage therapy for infections following hip or knee arthroplasty; these include case reports, retrospective studies, and prospective studies. The studies must provide detailed treatment protocols and outcome evaluations. Exclusion criteria: Studies of low relevance (e.g., those not involving the treatment of infections following hip or knee arthroplasty); animal experiments and laboratory studies; articles not peer-reviewed; studies that are incomplete or lack critical data; review articles and other types of publications.

### Data extraction and quality assessment

**Fig. 1:**
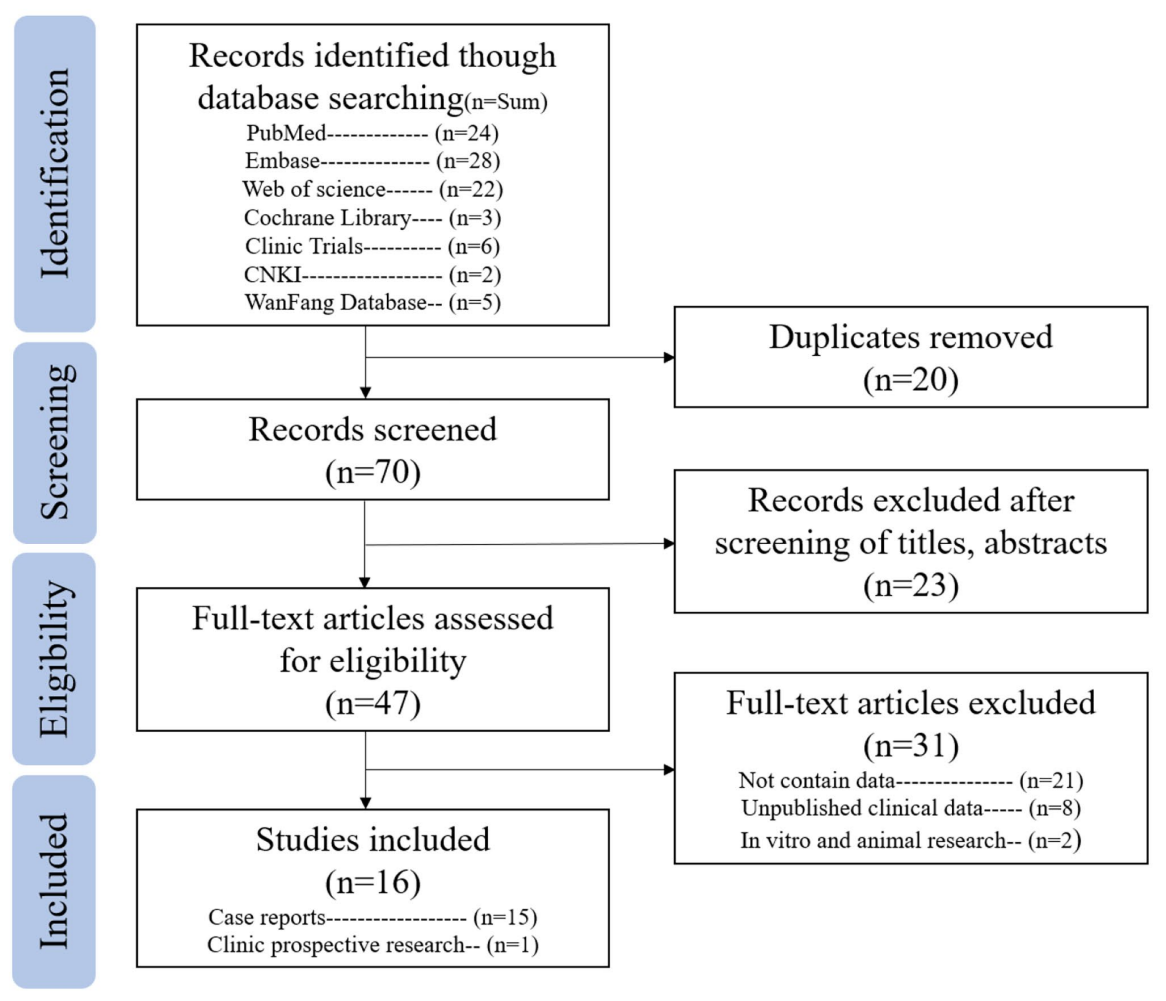
Flowchart showing the results of the search and the methodology selected

Key information was extracted from the included studies, including: study design (case reports, retrospective studies, prospective studies); the number of patients and their demographic characteristics; the type of infection and information on pathogens; treatment protocols (types of bacteriophages, routes of administration, dosages, and treatment duration); treatment outcomes (improvement in clinical symptoms, eradication of bacterial infection, and follow-up duration); and safety and adverse effects (complications). The search results were downloaded into Zotero 6.0 for evaluation. After removing duplicates, titles and abstracts were screened for eligibility. Full texts of the studies that met the eligibility criteria were reviewed and data were extracted. This culminated in the formation of this systematic review (Fig. 1).

## Results

### Studies characteristics (table 1)

A total of 16 studies were included, of which 15 were case reports and one was a prospective controlled study, involving a total of 42 patients. Among the included studies, six were from the USA, five from France, one from Germany, one from the Netherlands, one from Latvia, and one from Italy. The study participants were mostly patients over 60 years old, with an average age of 62.86 years. They had undergone hip or knee arthroplasty, with an average of 5.25 surgeries (calculated from reports that included surgical counts). The studies involved a total of 43 joints (29 hips and 14 knees), including one report where the same patient had infections in both the hip and knee on the same side. Details are provided in Table 1.

**Table 1:** Basic information of clinical studies on bacteriophage in PJI

No.	Author	Year	Country	Number of PJI patients	Patient’s Information						
					Gender	Age	Numbers of the surgery	Prosthetic joint	Date of the surgery	Bacteria	Co-morbidities
1	Ferry et al. [20]	2018	France	1	Female	80	6	Right Hip	2017	Multidrug-resistant *Pseudomonas aeruginosa* and Methicillin-susceptible *Staphylococcus aureus* (fully susceptible except for penicillin) from the swab of the pus. Then, operative samples confirmed MSSA in culture but not *P. aeruginosa*. And *Enterococcus faecalis* (susceptible to amoxicillin), *Staphylococcus lugdunensis* (susceptible to all antibiotics, including penicillin) were also detected.	diabetes mellitus type 2 and mild chronic kidney injury.
2	Patey et al. [21]	2018	France	3	Female	80	NR	Knee	2010	*Pseudomonas aeruginosa*	NR
					Female	90	NR	Hip	2010	Methicillin-resistant *Staphylococcus Aureus*, MRSA	NR
					Female	72	NR	Left Knee	2013	*Staphylococcus sp.*	NR
3	Tkhilaishvili et al. [22]	2019	Germany	1	Female	80	4	Right Knee	NR	Multi-drug resistant *Pseudomonas aeruginosa*	metabolic syndrome (diabetes mellitus type 2, obesity, essential hypertension) and chronic kidney failure.
4	Doub et al. [23]	2020	USA	1	Male	72	6	Right Knee	NR	Methicillin-resistant *Staphylococcus aureus*, MRSA	obesity and hyperlipidemia
5	Ferry et al. [24]	2020	France	3	Male	80	2	Left Knee	2015	Methicillin-susceptible *Staphylococcus aureus*, MSSA	parkinson disease, cardiac arrhythmia, hypertension,
					Male	84	5	Right Knee	2019	Methicillin-susceptible *Staphylococcus aureus*, MSSA	dyslipidemia
					Female	83	3	Right Knee	2019	Methicillin-susceptible *Staphylococcus aureus*, MSSA	hypertension and lymphoedema
6	Ferry et al. [25]	2020	France	1	Male	49	4	Right Knee	2016	Methicillin-susceptible *Staphylococcus aureus*, MSSA	NR
7	Cano et al. [26]	2021	USA	1	Male	62	14	Right Knee	2019	*Klebsiella pneumoniae* complex	obesity, diabetes mellitus
8	Doub et al. [27]	2021	USA	1	Female	79	5	Left Knee	NR	Multi-drug resistant *Staphylococcus epidermidis*	aplastic anemia
9	Ferry et al. [28]	2021	France	1	Male	88	2	Left Knee	NR	*Pseudomonas aeruginosa*	arrhythmia with severe cardiomyopathy
10	Neuts et al. [29]	2021	Netherlands	1	Male	76	8	Left Hip	2017	*Enterococcus faecalis*	None
11	Ramirez-Sanchez et al. [30]	2021	USA	1	Female	61	> 6	Right Knee	NR	Methicillin-susceptible *Staphylococcus aureus*, MSSA	NR
12	Schoeffel et al. [31]	2022	USA	1	Female	64	4 and 4	Right Hip and Knee	2021	Methicillin-resistant *Staphylococcus aureus*, MRSA	NR
13	Racenis et al. [32]	2022	Latvia	1	Male	21	9	Right Hip	2017	Multi-drug resistant *Pseudomonas aeruginosa*, Vancomycin-resistant *enterococci*, and *Staphylococcus epidermidis*	None
14	Cesta et al. [33]	2023	Italy	1	Female	62	3	Right Hip	2020	*Pseudomonas aeruginosa*	NR
15	Fedorov et al. [34]	2023	Russia	23	Female/Male	Avg: 56.0	NR	Hip	2012–2018	*Staphylococcus epidermidis*, MSSE: 8 *Staphylococcus epidermidis*, MRSE: 6 *Staphylococcus aureus*, MSSA: 8 *Staphylococcus aureus*, MRSA: 1	NR
16	Doub et al. [35]	2023	USA	1	Male	69	> 3	Left Knee	NR	*Enterococcus faecalis*	atrial fibrillation, diabetes and hypertension

### Sample description (table 2)

**Fig. 2:**
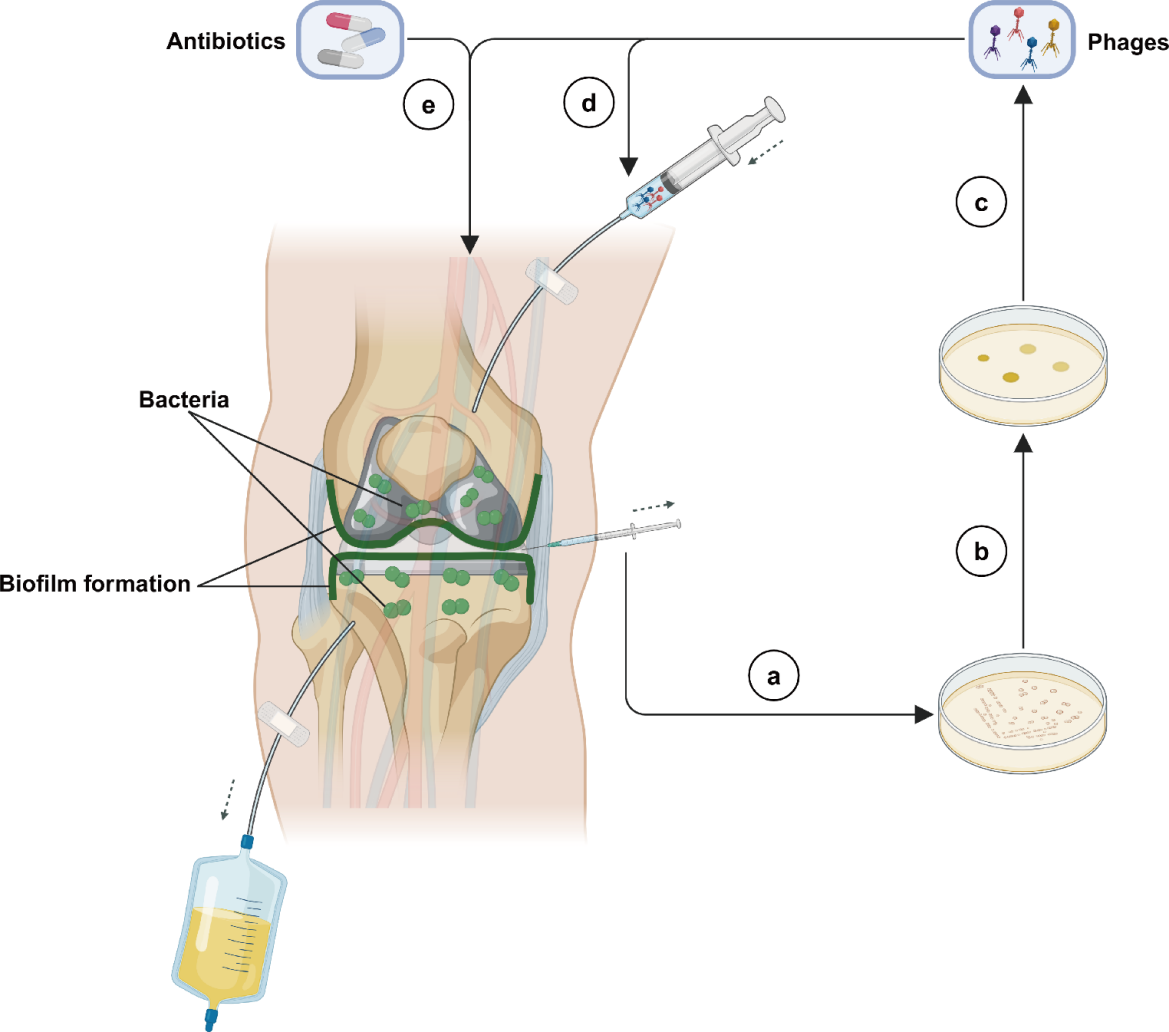
A schematic representation of the application of phage therapy techniques for PJI in the knee, the specific steps as follows: **a**: Isolate the patient’s pathogenic bacteria and cultivate them in vitro; **b**: Perform bacterial typing and screen for susceptible lytic bacteriophages; **c**: Amplify and remove endotoxins from the bacteriophages, and prepare qualified single or cocktail bacteriophage formulations according to Good Manufacturing Practice (GMP) standards; **d**: Select an appropriate treatment regimen based on the patient’s tolerance. The included studies reported methods such as intra-articular injection, drainage tube irrigation, local application with hydrogel and bone cement carriers, as well as systemic administration via oral or intravenous routes; **e**: Combine with antibiotic therapy

In all case reports, patients had a history of multiple surgeries, including debridement, one-stage revision, and two-stage revision arthroplasty. Among the infectious pathogens, the most common was *Staphylococcus aureus*, with a total of 18 cases, followed by *Staphylococcus epidermidis* with 16 cases, and *Pseudomonas aeruginosa* with five cases. Furthermore, regarding the use of bacteriophages, all reports specified the types of bacteriophages used for treatment. However, the details on bacteriophage types, timing of use, routes of administration, dosages, dosing frequencies, and duration of use varied among the reports. Of the 42 patients, 33 received bacteriophage cocktail therapy, and nine were treated with a single bacteriophage preparation. Additionally, all patients received suppressive antibiotic therapy postoperatively. Regarding routes of administration, some reports mentioned the use of intravenous injection or combined intra-articular administration. In some cases, bacteriophages were administered solely intra-articularly, either by direct injection into the joint cavity before wound closure during surgery or by continuous infusion through a drainage tube. Additionally, phage-loaded carrier the Defensive Antibacterial Coating (DAC^®^) hydrogel were applied to the prosthesis surface, and in some studies, oral administration was used. In the use of combination antibiotics, the types of antibiotics were adjusted in real-time based on the patient’s disease progression. Most case reports mentioned that patients received at least six weeks of suppressive antibiotic therapy postoperatively. The simplified treatment flowchart and specific methods included in these studies in Fig. 2; Table 2.

**Table 2:** Treatment strategies and prognosis

No.	Institution	Treatment				Complications	Follow up	Prognosis	Article Conclusion
		Single or Cocktail	Bacteriophage	Antibiotics	Surgery Plan				
1	The Croix-Rousse Hospital, Hospices Civils de Lyon	Cocktail (mixed the *P. aeruginosa* and the *S. aureus* phages in 2 different saline solutions of 10 mL as “compounded” drug products, local injection)	PP1493, PP1815, PP1957 (Pherecydes Pharma library)	SAT (850 mg/day daptomycin until month 3, then 6 g/day amoxicillin and 1800 mg/day clindamycin until month 6. Thereafter, only amoxicillin.)	DAIR (Changing the mobile parts of the prosthesis was not possible)	None	18 months	After a new DAIR procedure and ciprofloxacin was added 2 months, the outcome was favorable without any clinical signs of persistent infection.	The salvage use of a bacteriophage mix was safe and associated with a clinical success and a potential anti-biofilm activity in a patient with relapsing *S. aureus* PJI. Selecting the best bacteriophage mix based on a phagogram of the infecting strain should be performed before bacteriophage therapy.
2	The Villeneuve Saint Georges Hospital	Cocktail (Local injection)	Commercial bacteriophage suspension (Mainly from Microgen in Russia and the Eliava Institute in Georgia)	NR	NR	NR	NR	2012 *P. aeruginosa* clearance, but appearance of Enterococcus sp.	Phage therapy has much to offer, particularly for osteoarticular infections.
		Single (By flooding the infection site and via catheter in the 10 days following the operation)		NR	NR	NR	NR	2011 Complete cure, rapid recovery without recurrence after 1 year with retention of the hip prosthesis and osteosynthesis material in situ.	
		Single (By flooding the infection site)		NR	NR	NR	NR	2013 Initial partial disinfection with closure of several fistula followed by stabilization.	
3	Center for Musculoskeletal Surgery, Charité-Universitätsmedizin Berlin	Single (A single 100 ml loading dose of purified bacteriophage was applied locally during surgery, followed by administration of 5 ml of bacteriophage solution every 8 h through each of the four drains as a local delivery system for 5 days).	NR (the George Eliava Institute of Bacteriophages, Microbiology and Virology, Tbilisi, Georgia).	SAT (Intravenous treatment with colistin 150 mg every 24 h, meropenem 1 g every 12 h, and ceftazidime 2 g every 12 h. Discharged with oral rifampin 600 mg once daily and doxycycline 100 mg twice daily for 6 weeks.)	Prosthetic explanation and debridement	None	10 months	Two weeks after explanation of the prosthesis, debridement and exchange of the spacer was performed and four weeks later, reimplantation of the prosthesis was performed. Then, recovery is good.	Indicates a potential adjunctive role of phages for eradication of MDR biofilms with limited therapeutic options.
4	the University of Maryland Medical Center	Single (Two doses of IA bacteriophage (5.4 × 109 PFU) in 10 mL of normal saline (NS), daily IV bacteriophage (2.7 × 109 PFU in 50 mL of NS) was started the next day and discontinue after the third IV)	SaGR51Φ1 (Created by Adaptive Phage Therapeutics, APT)	SAT (IV daptomycin 1000 mg daily and continued for 6 weeks)	Explant of prosthesis components with placement of static vancomycin and tobramycin spacer.	Liver function abnormalities occurred during use the phage and recovered after discontinuation	NR	Intraoperative cultures were again negative and discharged in a week.	Bacteriophage therapy has tremendous potential to help cure PJIs, but phase 1 and 2 clinical trials need to be conducted.
5	The Croix-Rousse Hospital, Hospices Civils de Lyon	Cocktail (PhagoDAIR procedure)	PP1493, PP1815, PP1957 (the Pherecydes Pharma library)	SAT (Intravenous daptomycin and rifampin orally, followed by cotrimoxazole and clindamycin for a total duration of 3 months. After the relapse, added the pristinamycin. Added Cefalexin in the follow up.)	DAIR.	None	30 months	At 3 months, a new DAIR was performed, then, the outcome was favorable.	The PhagoDAIR procedure has the potential to be used as salvage for patients with relapsing S. aureus PKI, in combination with suppressive antibiotics to avoid considerable loss of function.
				SAT (Intravenous cefazolin and rifampin orally, after the DAIR cefazolin was switched to ofloxacin 3 weeks. Then, doxycycline was prescribed as suppressive therapy.)	DAIR	None	7 months	The outcome was favorable and no signs of infection, a negative C-reactive protein and pain-free walking.	
				SAT (Intravenous cloxacillin and rifampin orally, doxycycline was then prescribed as suppressive therapy.)	DAIR	None	11 months	At 4 months, a new DAIR was performed, but no superinfection was diagnosed.	
6	The Croix-Rousse Hospital, Hospices Civils de Lyon	Cocktail (300 mg of sterile DAC^®^ powder, with a solution of 5 ml sterile water for injection, and 1 ml of each bacteriophage was added)	PP1493, PP1815 (the Pherecydes phage bank)	SAT (Before the surgery, clindamycin was prescribed as suppressive therapy, after the surgery, intravenous with daptomycin 850 mg, one injection/day and tigecycline 100 mg as initial dose, followed by 50 mg injected every 12 h; After obtaining the results of the intraoperative bacterial culture, tigecycline was replaced with ceftazidime, ciprofloxacin, and rifampin)	DAIR + the DIEP free flap	None	12 months	After the phage was used, two DAIR were performed. After 1 year, a transfemoral amputation was performed.	Demonstrated the practical feasibility of the use of bacteriophages within a hydrogel to treat patients for knee megaprosthesis infection during a DAIR procedure. This is a potentially innovative approach to target the biofilm in patients with megaprosthesis knee infection.
7	Mayo Clinic	Single (Daily infusions of 6.3 × 10^10^ phages in 50 mL of normal saline intravenously each weekday for a total of 40 doses)	KpJH46Φ2 (the Adaptive Phage Therapeutics (APT) in Gaithersburg)	SAT (Vancomycin and rifampin and on oral cefadroxil in 2015. Then, oral penicillin in 2018. In 2019, added doxycycline, started on daptomycin and transitioned to oral linezolid; After 8 weeks of meropenem, transitioned to oral minocycline for life-long suppression.)	NO (The last surgery is incision and drainage, I&D)	None	8.5 months	Apparent resolution of symptoms and perform daily routine to an extent.	Phage therapy may be safe, effective, and well tolerated.
8	University of Maryland surgical infectious disease clinic	Single (2 × 10^10^ plaque forming units (PFU) of phage PM448 diluted in 10 mL of normal saline was injected into the intraarticular space)	PM448 (the PhagoMed in Austria)	SAT (Intravenous ertapenem 1 g daily and daptomycin 500 mg daily for 6 weeks. Then, transitioned to oral doxycycline 100 mg po bid. Oral rifampin started 1 week after DAIR and discontinued later.)	DAIR	The liver function showed a transient abnormality during the second day of surgery.	5 months	Full range of motion of knee and no clinical signs of recurrence.	The use of intraarticular bacteriophage therapy as an adjuvant to DAIR in recalcitrant PJIs holds promise to improve morbidity and reduce mortality.
9	The Croix-Rousse Hospital, Hospices Civils de Lyon	Cocktail (30 cc of the phage suspension was injected through the arthroscope.)	PP1450, PP1777, PP1792 (the Pherecydes Pharma library)	SAT (3 weeks of intravenous ceftazidime 6 g/day and oral ciprofloxacin 500 mg bid, at 6 months, the dose of ciprofloxacin was reduced to 250 mg bid.)	DAIR	None	12 months	The joint motion and walking were unpainful.	The PhagoDAIR procedure by arthroscopy has the potential to be used as salvage therapy for patients with P. aeruginosa relapsing PJI, in combination with suppressive antimicrobial therapy.
10	Sint Maartenskliniek	Cocktail (oral suspensio)	Pyophage and IntestiPhage (the Eliava Institute of Bacteriophage, Microbiology and Virology in Tbilisi, Georgia)	SAT (Teicoplanin 600 mg twice a day oral amoxicillin 1000 mg was administered 4 times a day, doxycycline 200 mg once a day. Then, it was reduced to 100 mg, oral amoxicillin 1,000 mg 4 times a day. During the second period, oral doxycycline 200 mg once a day.)	None	During the teicoplanin therapy, appeared kidney failure, then antibiotic changed, during the use of doxycycline, the presence of nausea, vomiting, and loss of appetite.	36 months	The patient had no hip complaints and no new cultures have been obtained.	As the first case on bacteriophage for an E. faecalis PJI. Demonstrated the safety and promising results for combination with antibiotics.
11	Department of Infectious Diseases and Global Public Health, University of California San Diego	Cocktail (the first cycle: one intra-articular dose followed by intravenous (IV) infusions every 12 h for 2 weeks, the Second cycle: a single intraoperative dose as well as IV treatments every 12 h for 6 weeks.)	the First cycle: AB-SA01 (J-Sa36, Sa83, Sa87. Ampliphi Biosciences, now Armata Pharmaceuticals, Marina Del Rey, U.S.) the Second cycle: SaGR51ø1 (Adaptive Phage Therapeutics, Gaithersburg, U.S.)	SAT (the first cycle: cefazolin 2 g IV every 8 h, 3 doses per day for 6 weeks. the Second cycle: concomitant IV cefazolin 2 g every 8 h for 6 weeks.)	Two-stage TKA	None	14 months	Bacterial culture is negative and weekly labs remained stable.	Phage as an adjunct to existing standard of care consisting of surgery and systemic antibiotics for the resolution of a recalcitrant MSSA PJI.
12	Joseph Medical Center	Single (10mL dose of bacteriophage therapy was injected per joint, Postoperative daily intravenous bacteriophage for 3 days.)	SaWIQ0488ø1 (Adaptive Phage Therapeutics, Gaithersburg, USA)	SAT (Postoperative daptomycin was continued for three more weeks, followed by Bactrim DS for three weeks)	One-stage exchange hip and knee	Slightly liver function abnormalities on the first postoperative day after phage treatment, but then did not deteriorate.	11 months	Since receiving bacteriophage therapy, there has been no evidence of recurrence, and the patient is ambulating without a cane, able to climb stairs and driving.	Used bacteriophage therapy as an adjuvant with surgical intervention to allow for the most successful application and consequently the best chance of cure.
13	Center of Nephrology, Pauls Stradins Clinical University Hospital, Riga, Latvia	Cocktail (Wound rinsing with 50 mL BFC 1.10 bacteriophage suspension was performed intraoperatively. During the first 7 days after surgery, with 40 mL (1 ml/min) of BFC 1.10 three times daily and then with 30 mL (1 ml/min) two times daily via an irrigation catheter for another 7 days.)	BFC 1.10 (Queen Astrid Military Hospital in Brussels, Belgium)	SAT (The cycle of antibiotic application is long and covers meropenem, colistin, piperacillin-tazobactam, linezolid, fluconazole, ceftazidime-avibactam, vancomycin, fosfomycin.)	Two-stage hip replacement	The patient suffered an acute kidney injury after two weeks of prior treatment with intravenous meropenem and colistin.	15 months	No local signs of infection, and radiography of the right hip did not reveal any signs of inflammation.	Demonstrates the possible use of bacteriophages and antibiotics in difficult-to-treat bone and soft tissue infections, where the additive effects of phages and antibiotics were observed.
14	Microbiology, Immunology, Infectious Diseases, and Transplants (MIMIT), University of Rome Tor Vergata, Rome, Italy	Single (I day 10 mL q8h, then 5 mL q8h via joint drainage for 2 weeks, before each application, 5 mL sodium bicarbonate (1.4%) was administered as previously described)	Pa53 (Eliava Institute in Tbilisi, Georgia)	SAT (ciprofloxacin 500 mg q8h 4 W, cephalosporin + ciprofloxacin, ceftolozane/tazobactam 1.5gr, q8h 2 W, cefepime 2gr q8h 4 W, meropenem 2gr q12h 12 W; daptomycin 500 mg q24h 2 W.)	DAIR + Mobile parts of change	After the first postoperative application of phage, high fever and chills appeared, which were relieved after the dose was reduced.	24 months	Good clinical conditions and no local signs of infection relapse were present.	Personalized Phage Therapy, in combination with meropenem, was found to be safe and effective in eradicating P. aeruginosa infection.
15	the Novosibirsk Research Institute of Traumatology and Orthopedics	Cocktail (6.0 mL of bacteriophage solution was added during mixing bone cement, 20.0 mL of bacteriophage was injected into the periprosthetic area daily for ten days through the drainage.)	Purified polyvalent pyobacteriophage, Sextaphage^®^ polyvalent pyobacteriophage, Staphylococcal bacteriophage (Microgen, Russia,)	SAT (In first two weeks, vancomycin was administered as an intravenous drip infusion, at a dose of 1.0 g twice a day, or cefazolin as an intravenous drip infusion at a dose of 2.0 g three times a day. After discharge, oral antibiotics: Ciprofloxacin + rifampicin or Trimethoprim/sulfamethoxazole to doxycycline.)	One-stage revision	2 patients with a febrile temperature after the phage preparation	12 months	One case appeared to relapse with PJI	Combination phage-antibiotic therapy is more effective than conventional antibiotic therapy and provides a significant advantage
16	Division of Clinical Care and Research, University of Maryland School of Medicine	Single (Not use in operation, received 1 × 1010 PFU/mL of bacteriophage diluted in 10mL of normal saline directly injected into knee with the use of an arthrocentesis for two days. Then, followed by intravenous bacteriophage therapy for 4 days in which 1 × 1010 PFU/mL were diluted in 50mL of normal saline and infused over 30 min.)	EF phage 1 (FDA IND’s 27513, University of Maryland, Baltimore)	SAT (piperacillin/tazobactam and levo floxacin, then changed to only intravenous ampicillin. After using phages, ampicillin was stopped and daily intravenous daptomycin 1 g daily was started for 7 days and then transitioned to oral amoxicillin 500 mg every 12 h. Then, intravenous vancomycin therapy for 6 weeks and then indefinite oral minocycline 100 mg twice a day.)	None	None	24 months	Without clinical signs of knee PJI recurrence, and PET/CT had no increased uptake.	Bacteriophage therapy for prosthetic joint infections has promise to reduce the morbidity that is associated with current treatments.

### Effectiveness and safety of treatment (table 2)

Patients included in the 16 studies were followed up for an average of 13.55 months (considering only reports with recorded follow-up durations), with two cases of recurrence observed. Among the 19 patients detailed in the case reports, one underwent amputation due to poor infection control one year post-surgery, while the others had favourable recovery outcomes. Statistical results from prospective clinical studies indicated that the overall response rate for patients treated with a combination of bacteriophages and antibiotics was 95.5%, with one patient experiencing infection recurrence during follow-up. Regarding adverse reactions, case reports documented one patient experiencing fever and chills; three patients developed liver function abnormalities attributed to bacteriophage therapy, with one patient recovering after discontinuation of treatment without life-threatening consequences. Additionally, two patients exhibited kidney function abnormalities, though these could not be definitively attributed to bacteriophage therapy alone due to concurrent antibiotic use. In the prospective controlled study, one patient was transferred to the control group due to evidence of other pathogens and a lack of bacteriophage titer in mid-sampling tests, which was considered a failure of bacteriophage therapy, while two patients experienced fever reactions.

### Synthesized analysis

Overall, the aggregated results from the included studies suggest that bacteriophage therapy is effective in eradicating infectious strains in various cases of prosthetic joint infections. Notably, it shows significant clinical efficacy against complex multi-drug-resistant bacteria. Compared to conventional antibiotic-only treatments, bacteriophage therapy is better tolerated, has fewer side effects, and lacks reports of severe adverse reactions on a large scale. This highlights its specific bactericidal mechanisms as a prominent advantage in combating multi-drug-resistant strains.

## Discussion

Our systematic review indicates that personalized bacteriophage therapy, grounded in modern biotechnological advances, serves as an effective adjunctive treatment for PJI. It not only demonstrates substantial clinical efficacy but also offers the advantage of a low-risk profile. Integrating observational analyses from various existing clinical studies on this treatment, we find that bacteriophage therapy is emerging as a critical adjunctive treatment, particularly in cases involving resistant complex bacteria or recurrent infections. It is increasingly indispensable in managing prosthetic joint infections. The following sections will provide a detailed discussion on these aspects:

In the treatment of PJI, one of the primary challenges is biofilm formation. Biofilms are highly organized polymeric structures composed of bacterial communities and extracellular matrix (ECM), adhering to surfaces of human tissues and implants. These structures are formed by the secretion of polysaccharides, proteins, lipids, and extracellular DNA (eDNA) [36]. Additionally, bacteria within the biofilm matrix can exist in various metabolic states, making it difficult to obtain accurate bacteriological evidence [37]. The physical separation of the biofilm and the varied states of the bacteria within pose significant challenges to conventional treatment [38]. Furthermore, bacteria can acquire antimicrobial resistance (AMR) through various mechanisms. Factors influencing bacterial resistance include overuse and misuse of antibiotics, which accelerate this process. Currently, the rate of increasing bacterial resistance surpasses the development of new antibiotics [39, 40]. According to the Global Antimicrobial Surveillance System (GLASS), antimicrobial resistance has been reported among 500,000 individuals across 22 countries. The severity of AMR is particularly pronounced in low- and middle-income countries due to inadequate surveillance, limited access to antibiotics, and insufficient laboratory capabilities [41]. These multiple factors collectively complicate the treatment of prosthetic joint infections with conventional antibiotics alone, often necessitating comprehensive, multidisciplinary interventions at medical centres.

Currently, the treatment guidelines and expert consensus for PJI emphasize a multidisciplinary approach involving orthopaedic surgeons, infectious disease specialists, internists, microbiologists, pharmacists, and rehabilitation physicians [42]. Treatment strategies are categorized based on the duration of clinical symptoms into acute and chronic infections. Acute infections may be managed with the Debridement, Antibiotics, and Implant Retention (DAIR) protocol, while chronic infections often require revision surgery (one/two stage revision) [43]. For refractory PJI or cases where joint reconstruction is unfeasible, alternative salvage procedures such as amputation, resection arthroplasty, and arthrodesis are considered [44]. Regardless of whether the infection is acute or chronic following primary replacement surgery, antibiotic therapy tailored to bacteriological evidence and the patient’s individual condition is an essential component of PJI management. Currently, research has compiled microbiological data on PJI, with Staphylococcus species (including *Staphylococcus* aureus and coagulase-negative *Staphylococci*) are the most common pathogens in PJI, accounting for approximately 40-60% of cases. Other Gram-positive pathogens (such as *Streptococci* and *Enterococci*) account for 10-20%, and Gram-negative bacilli for 5-20%. Moreover, the microbiological profile of infections varies between hip and knee prostheses due to differences in location and surgical techniques [45–47]. Joint aspiration and biopsy to obtain definitive bacteriological evidence are crucial for antibiotic selection. Systemic administration of antibiotics is indispensable for effective antibacterial treatment. However, most antibiotics cannot achieve sufficient local drug concentrations, necessitating their local application when required, which may include local injection, intra-articular catheter delivery, or combining with a carrier substance [48, 49]. In summary, a personalized approach to the selection of antibiotics, their administration routes, and treatment duration is advocated [50, 51]. In the coming years, knee and hip revision surgeries are projected to increase by 43–182%. This suggests that without improvements in current prevention and treatment strategies, the number of infections will likely rise [5]. Additionally, literature reports indicate that even with systematic SAT, the success rate is not 100%. Most patients receiving bacteriophage therapy are those for whom antibiotic treatments have failed. For these patients, bacteriophage therapy serves as an adjunct to both conservative and surgical treatments, aiming to enhance the success rate of suppressive antibiotic therapy [52, 53]. Therefore, it is essential and urgent to continue research and innovation in this therapeutic approach to address the ongoing challenges. This review, in screening clinical cases of treating PJI, found that in dealing with high treatment difficulty, the existence of multiple drug-resistant and recurrent PJI, and other complex cases, the trend of multiple medical institutions reusing bacteriophages has become increasingly apparent.

**Fig. 3:**
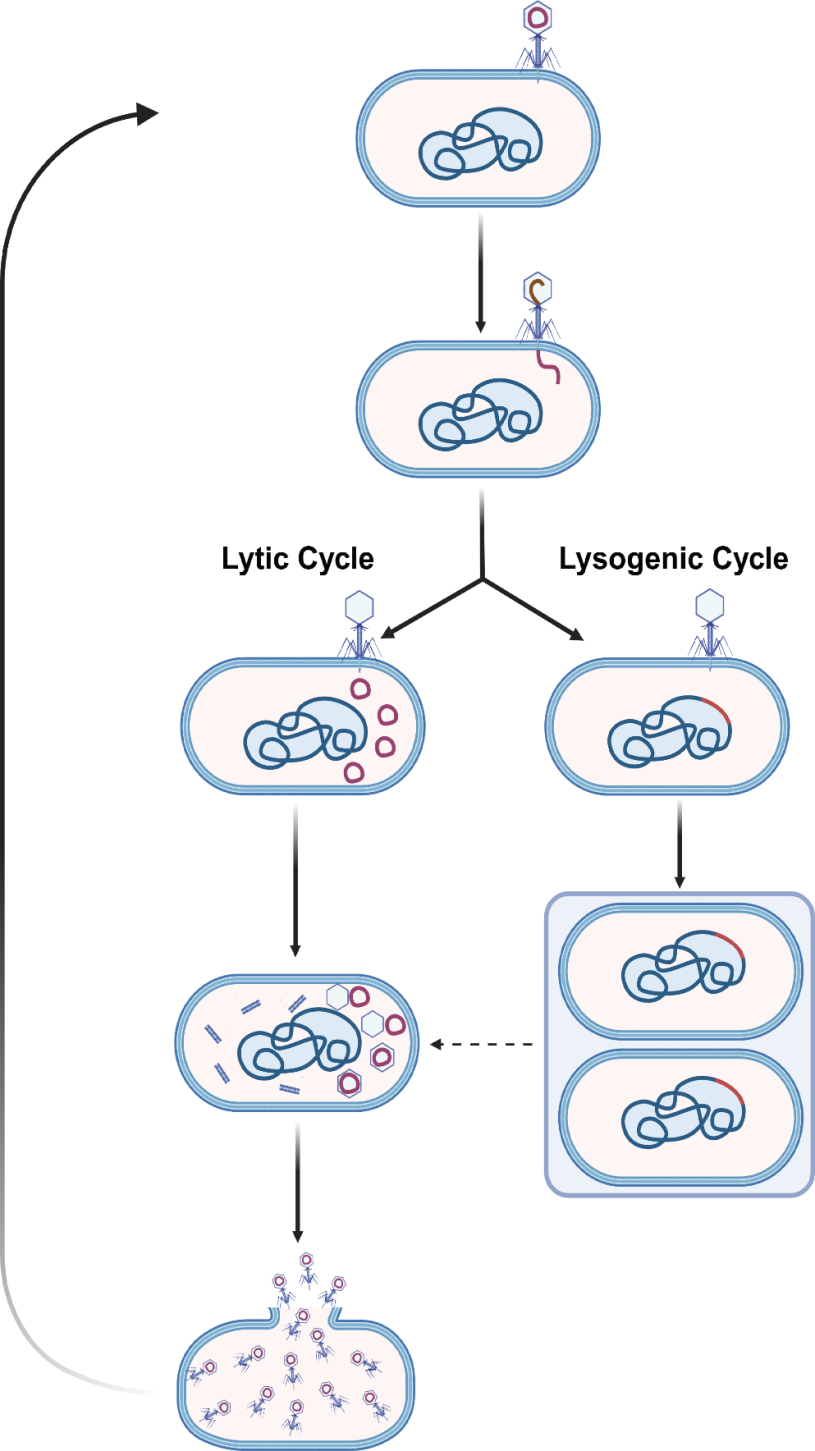
A schematic representation of the lytic cycle and lysogenic life cycle and the general processes of bacteriophages. Although the lysogenic cycle of temperate bacteriophages does not immediately cause bacterial lysis, it can induce genetic remodeling and, under suitable conditions, may transition into the lytic cycle, leading to bacterial destruction and replication of the bacteriophage. And the lytic cycle of virulent bacteriophages produces lysins that degrade the bacterial cell wall, rapidly leading to facilitates dissemination of themselves

Bacteriophages are abundantly present in natural environments and exhibit high specificity towards bacteria, making them of significant research interest. Regarding their mechanism of action, traditionally, it is believed that the primary mechanism of phages involves interacting with receptors on the host cell surface and using endolysins (peptidoglycan hydrolases) to inject their genome into the target bacteria. The replication method then depends on whether the phage is virulent or temperate. Virulent phages replicate through the lytic cycle, producing new phages while killing the bacteria, and temperate phages usually have two pathways: the lytic cycle and the lysogenic cycle. In the lysogenic cycle, the phage genome, known as a prophage, integrates with the host genome, replicating as part of the bacterial chromosome or as an independent plasmid. Under favorable conditions, the prophage can switch to the lytic cycle, releasing new phages and killing the host bacteria [54, 55] (Fig. 3). As research on phages has progressed, additional bactericidal mechanisms have been discovered, such as reducing biofilm surface polymers via enzymatic action, lowering bacterial virulence, and assisting the host immune system in bacterial clearance [56–58]. Bacteriophages can also intervene in bacterial dissemination by expressing phage-carried sporulation genes during infection, affecting the formation of bacterial spores to counteract bacterial defense mechanisms mediated by dormancy, thereby intervening in bacterial spread [59]. In the clinical studies included in this review, bacteriophage therapy demonstrated significant efficacy to control the disease, with 39 out of 42 patients showing substantial symptom relief. Regarding treatment safety, three patients experienced adverse reactions such as fever and chills. Overall, these adverse reactions were relatively mild, and they alleviated after reducing or discontinuing bacteriophage treatment. These reactions are likely due to potential bacterial residual cell wall component into the phage preparation, or could be due to bacterial lysis in vivo or to the host’s immune response. These mechanisms require further readership to clearly understand the pathophysiology of such symptoms.

Of course, bacteriophage therapy also has certain limitations. Its highly specific mechanism of action is like a double-edged sword. Each type of phage has a host range and is only effective against specific bacterial strains. This specificity means not all phages are suitable for treating PJI. Therefore, clinical phage preparations require accurate bacteriological evidence from the patient to ensure the selected phage can lyse the target bacteria. This requirement restricts the scalability of standardized phage preparations [60]. Additionally, studies have shown that bacteria can develop resistance to phages by altering or suppressing the expression of their receptors [61]. From the results of this review, several limitations are evident. First, there is a predominance of case studies, with few large-scale clinical trials. This raises questions about the ability to statistically evaluate and describe the combined results. The differences in study types may also introduce biases in assessing clinical efficacy and adverse events. Second, all patients received standardized antibiotic therapy alongside phage treatment. Antibiotics and phages may have synergistic effects. Additionally, in some studies, patients underwent surgical treatment concurrent with bacteriophage therapy. These factors could confound the assessment of bacteriophage therapy’s efficacy. Furthermore, in most case reports, there is a lack of uniform standards regarding the source, formulation, drug concentration and dosage, administration route, administration frequency, and treatment duration of bacteriophages, the lack of standardization makes it difficult to draw definitive conclusions. Although the results of this review do not differ significantly from previously published systematic reviews, caution is still needed in evaluating and confirming these data due to the lack of large-scale clinical experiments and standardized experimental designs.

Recently, several new phage research clinical teams have been established in Europe. In Belgium, PHAGEFORCE, a multidisciplinary initiative, has been established by the “Multidisciplinary Phage Task Force” to standardize bacteriophage therapy and prospectively collect data. In France, the “PHAGEinLYON” clinic program has also been established to provide pharmaceutical-grade phages to patients with severe infections and systematically collect treatment metrics [13]. Additionally, new concepts for phage treatment of PJI are being implemented. For example, the Center of Reference for Infection of Osteoarticular Complexes (CRIOAc) at the Croix-Rousse Hospital has innovatively proposed the concept of “PhagoDAIR” which involves injecting a cocktail of active bacteriophages during open or arthroscopic DAIR surgery, with promising clinical outcomes [21, 25]. Currently, various antibacterial methods inspired by bacteriophages, including bacteriophages themselves, their enzymes and derivatives, effects mediating biofilm destruction, and enhancing antibiotic sensitivity, may lead to more commercialized products. Despite our limited understanding of most bacteriophage functions, the potential of this vast field remains immense. Regarding clinical research, given the potential efficacy of bacteriophage therapy for refractory PJI, larger-scale clinical controlled studies should be conducted according to current clinical practice guidelines to support the safety and effectiveness of bacteriophage therapy. In the future, as the limitations of conventional treatments become more apparent and foundational research and clinical applications of bacteriophage therapy progress, new discoveries are likely to emerge, facilitating the clinical translation of bacteriophage therapy and ushering in a new era for the treatment of PJI.

## Conclusions

Bacteriophage therapy has demonstrated good efficacy in various complex infection cases, particularly those caused by antibiotic-resistant strains. Given the growing issue of antibiotic resistance, its specificity and low side effects make bacteriophage therapy a promising alternative treatment. However, factors such as the type of infection, the patient’s underlying conditions, and the treatment regimen can also affect the efficacy of bacteriophage therapy. Additionally, the small sample sizes of current studies, along with inconsistencies in bacteriophage sources, preparation methods, and administration routes, may also affect the outcomes. Future research requires standardized phage formulations and the inclusion of large sample sizes, randomized controlled trials, and prospective studies to further explore their efficacy and mechanisms.

## Electronic supplementary material

Below is the link to the electronic supplementary material.

Supplementary Material 1

## References

[1] Kamath AF, Ong KL, Lau E et al (2015) Quantifying the Burden of Revision Total Joint Arthroplasty for Periprosthetic Infection. J Arthroplasty 30:1492–1497. 10.1016/j.arth.2015.03.035 25865815 10.1016/j.arth.2015.03.035

[2] Sangaletti R, Zanna L, Akkaya M et al (2023) Periprosthetic joint infection in patients with multiple arthroplasties. Bone Joint J 105–B:294–300. 10.1302/0301-620X.105B3.BJJ-2022-0800.R1 36854322 10.1302/0301-620X.105B3.BJJ-2022-0800.R1

[3] Zhao H, Li L, Wang H-Y et al (2024) Efficacy analysis of clinical serological indicators in the diagnosis of postoperative periprosthetic joint infection in patients with rheumatoid arthritis or osteoarthritis. Int Orthop 48:1945–1952. 10.1007/s00264-024-06171-y 38581467 10.1007/s00264-024-06171-y

[4] Yang C, Ji B, Li G et al (2024) Ninety-day postoperative mortality and complications in continuous and unselected single-stage revisions for chronic periprosthetic joint infection. Int Orthop 48:1691–1700. 10.1007/s00264-024-06152-1 38526615 10.1007/s00264-024-06152-1

[5] Piuzzi N, Klika A, Lu Q et al (2024) Periprosthetic joint infection and immunity: current understanding of host-microbe interplay. J Orthop Res 42:7–20. 10.1002/jor.25723 37874328 10.1002/jor.25723

[6] Premkumar A, Kolin DA, Farley KX et al (2021) Projected Economic Burden of Periprosthetic Joint Infection of the hip and knee in the United States. J Arthroplasty 36:1484–1489e3. 10.1016/j.arth.2020.12.005 33422392 10.1016/j.arth.2020.12.005

[7] Taha M, Abdelbary H, Ross F, Carli A (2018) New innovations in the treatment of PJI and Biofilms-Clinical and preclinical topics. Curr Rev Musculoskelet Med 11:380–388. 10.1007/s12178-018-9500-5 29926287 10.1007/s12178-018-9500-5 PMC6105481

[8] Köhler T, Luscher A, Falconnet L et al (2023) Personalized aerosolised bacteriophage treatment of a chronic lung infection due to multidrug-resistant Pseudomonas aeruginosa. Nat Commun 14:3629. 10.1038/s41467-023-39370-z 37369702 10.1038/s41467-023-39370-z PMC10300124

[9] Leitner L, Ujmajuridze A, Chanishvili N et al (2021) Intravesical bacteriophages for treating urinary tract infections in patients undergoing transurethral resection of the prostate: a randomised, placebo-controlled, double-blind clinical trial. Lancet Infect Dis 21:427–436. 10.1016/S1473-3099(20)30330-3 32949500 10.1016/S1473-3099(20)30330-3

[10] Johri AV, Johri P, Hoyle N et al (2023) Case report: successful treatment of recurrent E. Coli infection with bacteriophage therapy for patient suffering from chronic bacterial prostatitis. Front Pharmacol 14:1243824. 10.3389/fphar.2023.1243824 37790805 10.3389/fphar.2023.1243824 PMC10544980

[11] Karn SL, Bhartiya SK, Pratap A et al (2024) A Randomized, Placebo-controlled, double-blind clinical trial of bacteriophage cocktails in chronic wound infections. Int J Low Extrem Wounds 15347346231226342. 10.1177/15347346231226342 10.1177/15347346231226342 38233034

[12] Khanal D, Chang RYK, Hick C et al (2021) Enteric-coated bacteriophage tablets for oral administration against gastrointestinal infections. Int J Pharm 609:121206. 10.1016/j.ijpharm.2021.121206 34673163 10.1016/j.ijpharm.2021.121206

[13] Ferry T, Onsea J, Roussel-Gaillard T et al (2024) Bacteriophage therapy in musculoskeletal infections: from basic science to clinical application. EFORT Open Rev 9:339–348. 10.1530/EOR-24-0042 38726986 10.1530/EOR-24-0042 PMC11099583

[14] Uyttebroek S, Chen B, Onsea J et al (2022) Safety and efficacy of phage therapy in difficult-to-treat infections: a systematic review. Lancet Infect Dis 22:e208–e220. 10.1016/S1473-3099(21)00612-5 35248167 10.1016/S1473-3099(21)00612-5

[15] Suster K, Cör A (2022) Fast and specific detection of staphylococcal PJI with bacteriophage-based methods within 104 sonicate fluid samples. J Orthop Res 40:1358–1364. 10.1002/jor.25167 34432330 10.1002/jor.25167

[16] DePalma B, Nandi S, Chaudhry W et al (2022) Assessment of Staphylococcal Clinical isolates from Periprosthetic Joint Infections for potential bacteriophage therapy. J BONE JOINT SURGERY-AMERICAN VOLUME 104:693–699. 10.2106/JBJS.21.00958 10.2106/JBJS.21.00958 35167506

[17] Kaur S, Harjai K, Chhibber S (2016) In vivo Assessment of Phage and Linezolid Based Implant Coatings for Treatment of Methicillin Resistant S. aureus (MRSA) mediated Orthopaedic device related infections. 10.1371/journal.pone.0157626. PLOS ONE 11:10.1371/journal.pone.0157626 PMC4917197 27333300

[18] Sosa B, Niu V, Turajane K et al (2020) 2020 John Charnley Award: the antimicrobial potential of bacteriophage-derived lysin in a murine debridement, antibiotics, and implant retention model of prosthetic joint infection. BONE JOINT J 102B:3–10. 10.1302/0301-620X.102B7.BJJ-2019-1590.R1 10.1302/0301-620X.102B7.BJJ-2019-1590.R1 32600192

[19] Moher D, Liberati A, Tetzlaff J, Altman DG (2009) Preferred reporting items for systematic reviews and meta-analyses: the PRISMA statement. PLoS Med 6:e1000097. 10.1371/journal.pmed.1000097 19621072 10.1371/journal.pmed.1000097 PMC2707599

[20] Ferry T, Leboucher G, Fevre C et al (2018) Salvage Debridement, Antibiotics and Implant Retention (DAIR) with local injection of a selected cocktail of bacteriophages: is it an option for an Elderly Patient with Relapsing Staphylococcus aureus Prosthetic-Joint infection? Open Forum Infect Dis 5:ofy269. 10.1093/ofid/ofy269 30474047 10.1093/ofid/ofy269 PMC6240628

[21] Patey O, McCallin S, Mazure H et al (2018) Clinical indications and compassionate use of phage therapy: personal experience and literature review with a focus on Osteoarticular infections. 10.3390/v11010018. Viruses 11:10.3390/v11010018 PMC6356659 30597868

[22] Tkhilaishvili T, Winkler T, Müller M et al (2019) Bacteriophages as adjuvant to antibiotics for the Treatment of Periprosthetic Joint Infection caused by Multidrug-Resistant Pseudomonas aeruginosa. Antimicrob Agents Chemother 64. 10.1128/AAC.00924-19 10.1128/AAC.00924-19 PMC7187616 31527029

[23] Doub JB, Ng VY, Johnson AJ et al (2020) Salvage bacteriophage therapy for a chronic MRSA prosthetic joint infection. Antibiotics 9. 10.3390/antibiotics9050241 10.3390/antibiotics9050241 PMC7277870 32397354

[24] Ferry T, Kolenda C, Batailler C et al (2020) Phage therapy as adjuvant to conservative surgery and antibiotics to salvage patients with relapsing S. Aureus prosthetic knee infection. Front Med 7. 10.3389/fmed.2020.570572 10.3389/fmed.2020.570572 PMC7701306 33304911

[25] Ferry T, Batailler C, Petitjean C et al (2020) The potential innovative use of bacteriophages within the DAC(^®^) hydrogel to treat patients with knee megaprosthesis infection requiring Debridement antibiotics and Implant Retention and Soft tissue Coverage as Salvage Therapy. Front Med (Lausanne) 7:342. 10.3389/fmed.2020.00342 32850878 10.3389/fmed.2020.00342 PMC7410981

[26] Cano EJ, Caflisch KM, Bollyky PL et al (2021) Phage therapy for limb-threatening prosthetic knee Klebsiella pneumoniae infection: Case Report and in Vitro characterization of anti-biofilm activity. Clin Infect Dis 73:E144–E151. 10.1093/cid/ciaa705 32699879 10.1093/cid/ciaa705 PMC8246933

[27] Doub JB, Ng VY, Wilson E et al (2021) Successful treatment of a recalcitrant staphylococcus epidermidis prosthetic knee infection with intraoperative bacteriophage therapy. Pharmaceuticals 14. 10.3390/ph14030231 10.3390/ph14030231 PMC7998749 33800146

[28] Ferry T, Kolenda C, Batailler C et al (2021) Case Report: arthroscopic debridement antibiotics and Implant Retention with Local Injection of Personalized Phage Therapy to Salvage a Relapsing Pseudomonas Aeruginosa prosthetic knee infection. Front Med (Lausanne) 8:569159. 10.3389/fmed.2021.569159 34026768 10.3389/fmed.2021.569159 PMC8132876

[29] Neuts A-S, Berkhout HJ, Hartog A, Goosen JHM (2021) Bacteriophage therapy cures a recurrent Enterococcus faecalis infected total hip arthroplasty? A case report. Acta Orthop 92:678–680. 10.1080/17453674.2021.1968714 34415227 10.1080/17453674.2021.1968714 PMC8635569

[30] Ramirez-Sanchez C, Gonzales F, Buckley M et al (2021) Successful treatment of staphylococcus aureus prosthetic joint infection with bacteriophage therapy. Viruses 13. 10.3390/v13061182 10.3390/v13061182 PMC8233819 34205687

[31] Schoeffel J, Wang EW, Gill D et al (2022) Successful use of salvage bacteriophage therapy for a recalcitrant MRSA knee and hip prosthetic joint infection. Pharmaceuticals 15. 10.3390/ph15020177 10.3390/ph15020177 PMC8877365 35215290

[32] Racenis K, Rezevska D, Madelane M et al (2022) Use of phage cocktail BFC 1.10 in Combination with Ceftazidime-Avibactam in the treatment of Multidrug-Resistant Pseudomonas aeruginosa Femur Osteomyelitis—A Case Report. Front Med 9. 10.3389/fmed.2022.851310 10.3389/fmed.2022.851310 PMC9081798 35547216

[33] Cesta N, Pini M, Mulas T et al (2023) Application of phage therapy in a case of a chronic hip-prosthetic joint infection due to Pseudomonas aeruginosa: an Italian real-life experience and in Vitro Analysis. Open Forum Infect Dis 10. 10.1093/ofid/ofad051 10.1093/ofid/ofad051 PMC9969736 36861092

[34] Fedorov E, Samokhin A, Kozlova Y et al (2023) Short-term outcomes of phage-antibiotic combination treatment in adult patients with periprosthetic hip joint infection. Viruses 15. 10.3390/v15020499 10.3390/v15020499 PMC9964274 36851713

[35] Doub JB, Chan B, Johnson AJ (2023) Salphage: salvage bacteriophage therapy for a chronic Enterococcus faecalis prosthetic joint infection. IDCases 33:e01854. 10.1016/j.idcr.2023.e01854 37577050 10.1016/j.idcr.2023.e01854 PMC10413068

[36] Limoli DH, Jones CJ, Wozniak DJ (2015) Bacterial extracellular polysaccharides in Biofilm formation and function. 10.1128/microbiolspec.MB-0011-2014. Microbiol Spectr 3:10.1128/microbiolspec.MB-0011-2014 PMC4657554 26185074

[37] Visperas A, Santana D, Klika AK et al (2022) Current treatments for biofilm-associated periprosthetic joint infection and new potential strategies. J Orthop Res 40:1477–1491. 10.1002/jor.25345 35437846 10.1002/jor.25345 PMC9322555

[38] Molina-Manso D, del Prado G, Ortiz-Pérez A et al (2013) In vitro susceptibility to antibiotics of staphylococci in biofilms isolated from orthopaedic infections. Int J Antimicrob Agents 41:521–523. 10.1016/j.ijantimicag.2013.02.018 23611308 10.1016/j.ijantimicag.2013.02.018

[39] Abushaheen MA, Muzaheed, Fatani AJ et al (2020) Antimicrobial resistance, mechanisms and its clinical significance. Dis Mon 66:100971. 10.1016/j.disamonth.2020.100971 32201008 10.1016/j.disamonth.2020.100971

[40] Ghosh D, Veeraraghavan B, Elangovan R, Vivekanandan P (2020) Antibiotic resistance and epigenetics: more to it than meets the Eye. Antimicrob Agents Chemother 64. 10.1128/AAC.02225-19 10.1128/AAC.02225-19 PMC6985748 31740560

[41] (2022) Global Antimicrobial Resistance and Use Surveillance System (GLASS) report 2022. World Health Organization

[42] Walter N, Rupp M, Baertl S et al (2022) Periprosthetic joint infection: patients benefit from a multidisciplinary team approach. Bone Joint Res 11:8–9. 10.1302/2046-3758.111.BJR-2021-0499 34994575 10.1302/2046-3758.111.BJR-2021-0499 PMC8801164

[43] Kapadia BH, Berg RA, Daley JA et al (2016) Periprosthetic joint infection. Lancet 387:386–394. 10.1016/S0140-6736(14)61798-0 26135702 10.1016/S0140-6736(14)61798-0

[44] Leitner L, Posch F, Amerstorfer F et al (2020) The Dark side of Arthroplasty: competing risk analysis of failed hip and knee arthroplasty with Periprosthetic Joint infection. J Arthroplasty 35:2601–2606e1. 10.1016/j.arth.2020.04.078 32451282 10.1016/j.arth.2020.04.078

[45] Triffault-Fillit C, Ferry T, Laurent F et al (2019) Microbiologic epidemiology depending on time to occurrence of prosthetic joint infection: a prospective cohort study. Clin Microbiol Infect 25:353–358. 10.1016/j.cmi.2018.04.035 29803842 10.1016/j.cmi.2018.04.035

[46] Manning L, Metcalf S, Clark B et al (2020) Clinical characteristics, etiology, and initial management strategy of newly diagnosed Periprosthetic Joint infection: a Multicenter, prospective observational cohort study of 783 patients. Open Forum Infect Dis 7:ofaa068. 10.1093/ofid/ofaa068 32432148 10.1093/ofid/ofaa068 PMC7224250

[47] Tsai Y, Chang C-H, Lin Y-C et al (2019) Different microbiological profiles between hip and knee prosthetic joint infections. J Orthop Surg (Hong Kong) 27:2309499019847768. 10.1177/2309499019847768 31117922 10.1177/2309499019847768

[48] Gonzalez Moreno M, Trampuz A, Di Luca M (2017) Synergistic antibiotic activity against planktonic and biofilm-embedded Streptococcus agalactiae, Streptococcus pyogenes and Streptococcus oralis. J Antimicrob Chemother 72:3085–3092. 10.1093/jac/dkx265 28961884 10.1093/jac/dkx265

[49] Steadman W, Chapman P, Schuetz M et al (2023) Local antibiotic delivery options in prosthetic joint infection. 10.3390/antibiotics12040752. ANTIBIOTICS-BASEL 12:10.3390/antibiotics12040752 PMC10134995 37107114

[50] Miller R, Higuera CA, Wu J et al (2020) Periprosthetic Joint Infection: a review of Antibiotic Treatment. JBJS Rev 8:e1900224. 10.2106/JBJS.RVW.19.00224 32678538 10.2106/JBJS.RVW.19.00224

[51] Nelson SB, Pinkney JA, Chen AF, Tande AJ (2023) Periprosthetic Joint infection: current clinical challenges. Clin Infect Dis 77:e34–e45. 10.1093/cid/ciad360 37434369 10.1093/cid/ciad360 PMC11004930

[52] Prendki V, Ferry T, Sergent P et al (2017) Prolonged suppressive antibiotic therapy for prosthetic joint infection in the elderly: a national multicentre cohort study. Eur J Clin Microbiol Infect Dis 36:1577–1585. 10.1007/s10096-017-2971-2 28378243 10.1007/s10096-017-2971-2

[53] Escudero-Sanchez R, Senneville E, Digumber M et al (2020) Suppressive antibiotic therapy in prosthetic joint infections: a multicentre cohort study. Clin Microbiol Infect 26:499–505. 10.1016/j.cmi.2019.09.007 31539638 10.1016/j.cmi.2019.09.007

[54] Salmond GPC, Fineran PC (2015) A century of the phage: past, present and future. Nat Rev Microbiol 13:777–786. 10.1038/nrmicro3564 26548913 10.1038/nrmicro3564

[55] Dy RL, Richter C, Salmond GPC, Fineran PC (2014) Remarkable mechanisms in microbes to resist phage infections. Annu Rev Virol 1:307–331. 10.1146/annurev-virology-031413-085500 26958724 10.1146/annurev-virology-031413-085500

[56] Roach DR, Donovan DM (2015) Antimicrobial bacteriophage-derived proteins and therapeutic applications. Bacteriophage 5:e1062590. 10.1080/21597081.2015.1062590 26442196 10.1080/21597081.2015.1062590 PMC4590002

[57] Shahed-Al-Mahmud M, Roy R, Sugiokto FG et al (2021) Phage φAB6-Borne depolymerase combats Acinetobacter baumannii Biofilm formation and infection. Antibiot (Basel) 10. 10.3390/antibiotics10030279 10.3390/antibiotics10030279 PMC7998257 33803296

[58] Kolenda C, Josse J, Medina M et al (2020) Evaluation of the activity of a combination of three bacteriophages alone or in Association with antibiotics on Staphylococcus aureus embedded in Biofilm or internalized in Osteoblasts. Antimicrob Agents Chemother 64. 10.1128/AAC.02231-19 10.1128/AAC.02231-19 PMC7038305 31871084

[59] Schwartz DA, Rodríguez-Ramos JA, Shaffer M et al (2023) Human-gut Phages Harbor Sporulation genes. mBio 14:e0018223. 10.1128/mbio.00182-23 37042671 10.1128/mbio.00182-23 PMC10294663

[60] Huang Y, Wang W, Zhang Z et al (2022) Phage products for fighting Antimicrobial Resistance. 10.3390/microorganisms10071324. Microorganisms 10:10.3390/microorganisms10071324 PMC9324367 35889048

[61] Garb J, Lopatina A, Bernheim A et al (2022) Multiple phage resistance systems inhibit infection via SIR2-dependent NAD(+) depletion. Nat Microbiol 7:1849–1856. 10.1038/s41564-022-01207-8 36192536 10.1038/s41564-022-01207-8

