# Supplementary material for: Bacteriophage therapy as an innovative strategy for the treatment of Periprosthetic Joint Infection: a systematic review
Source: Int Orthop. 2024 Sep 10;48(11):2809–25. doi: 10.1007/s00264-024-06295-1 (PMC11490438; doi:10.1007/s00264-024-06295-1)
Supplement: Supplementary file 1 — Supplementary Material 1 [file 264_2024_6295_MOESM1_ESM.docx]

**Supplementary Material：search strategy**

**Hip Arthroplasty**

Pubmed：((((((((((((((((((("Arthroplasty, Replacement, Hip"[Mesh]) OR (Arthroplasties, Replacement, Hip)) OR (Arthroplasty, Hip Replacement)) OR (Hip Replacement Arthroplasties)) OR (Hip Prosthesis Implantation)) OR (Hip Prosthesis Implantations)) OR (Implantation, Hip Prosthesis)) OR (Prosthesis Implantation, Hip)) OR (Replacement Arthroplasties, Hip)) OR (Replacement Arthroplasty, Hip)) OR (Arthroplasties, Hip Replacement)) OR (Hip Replacement Arthroplasty)) OR (Hip Replacement, Total)) OR (Replacement, Total Hip)) OR (Total Hip Replacements)) OR (Total Hip Replacement)) OR (Total Hip Arthroplasty)) OR (Arthroplasty, Total Hip)) OR (Hip Arthroplasty, Total)) OR (Total Hip Arthroplasties)

Embase：'hip arthroplasty'/exp OR 'hip arthroplasty' OR 'arthroplasties, replacement, hip' OR 'arthroplasty, hip replacement' OR 'hip replacement arthroplasties' OR 'hip prosthesis implantation' OR 'hip prosthesis implantations' OR 'implantation, hip prosthesis' OR 'prosthesis implantation, hip' OR 'replacement arthroplasties, hip' OR 'replacement arthroplasty, hip' OR 'arthroplasties, hip replacement' OR 'hip replacement arthroplasty' OR 'hip replacement, total' OR 'replacement, total hip' OR 'total hip replacements' OR 'total hip replacement' OR 'total hip arthroplasty' OR 'arthroplasty, total hip' OR 'hip arthroplasty, total' OR 'total hip arthroplasties'

Web of science：(((((((((((((((((((ALL=(Arthroplasty, Replacement, Hip)) OR ALL=(Arthroplasties, Replacement, Hip)) OR ALL=(Arthroplasty, Hip Replacement)) OR ALL=(Hip Replacement Arthroplasties)) OR ALL=(Hip Prosthesis Implantation)) OR ALL=(Hip Prosthesis Implantations)) OR ALL=(Implantation, Hip Prosthesis)) OR ALL=(Prosthesis Implantation, Hip)) OR ALL=(Replacement Arthroplasties, Hip)) OR ALL=(Replacement Arthroplasty, Hip)) OR ALL=(Arthroplasties, Hip Replacement)) OR ALL=(Hip Replacement Arthroplasty)) OR ALL=(Hip Replacement, Total)) OR ALL=(Replacement, Total Hip)) OR ALL=(Total Hip Replacements)) OR ALL=(Total Hip Replacement)) OR ALL=(Total Hip Arthroplasty)) OR ALL=(Arthroplasty, Total Hip)) OR ALL=(Hip Arthroplasty, Total)) OR ALL=(Total Hip Arthroplasties)

**Knee Arthroplasty**

Pubmed：((((((((((((((((((((((((((((((("Arthroplasty, Replacement, Knee"[Mesh]) OR (Arthroplasties, Replacement, Knee)) OR (Arthroplasty, Knee Replacement)) OR (Knee Replacement Arthroplasties)) OR (Knee Replacement Arthroplasty)) OR (Replacement Arthroplasties, Knee)) OR (Knee Arthroplasty, Total)) OR (Arthroplasty, Total Knee)) OR (Total Knee Arthroplasty)) OR (Replacement, Total Knee)) OR (Total Knee Replacement)) OR (Knee Replacement, Total)) OR (Knee Arthroplasty)) OR (Arthroplasty, Knee)) OR (Arthroplasties, Knee Replacement)) OR (Replacement Arthroplasty, Knee)) OR (Arthroplasty, Replacement, Partial Knee)) OR (Unicompartmental Knee Arthroplasty)) OR (Arthroplasty, Unicompartmental Knee)) OR (Knee Arthroplasty, Unicompartmental)) OR (Unicondylar Knee Arthroplasty)) OR (Arthroplasty, Unicondylar Knee)) OR (Knee Arthroplasty, Unicondylar)) OR (Partial Knee Arthroplasty)) OR (Arthroplasty, Partial Knee)) OR (Knee Arthroplasty, Partial)) OR (Unicondylar Knee Replacement)) OR (Knee Replacement, Unicondylar)) OR (Partial Knee Replacement)) OR (Knee Replacement, Partial)) OR (Unicompartmental Knee Replacement)) OR (Knee Replacement, Unicompartmental)

Embase：'knee arthroplasty'/exp OR 'knee arthroplasty' OR 'Arthroplasties, Replacement, Knee' OR 'Arthroplasty, Knee Replacement' OR 'Knee Replacement Arthroplasties' OR 'Knee Replacement Arthroplasty' OR 'Replacement Arthroplasties, Knee' OR 'Knee Arthroplasty, Total' OR 'Arthroplasty, Total Knee' OR 'Total Knee Arthroplasty' OR 'Replacement, Total Knee' OR 'Total Knee Replacement' OR 'Knee Replacement, Total' OR 'Knee Arthroplasty' OR 'Arthroplasty, Knee' OR 'Arthroplasties, Knee Replacement' OR 'Replacement Arthroplasty, Knee' OR 'Arthroplasty, Replacement, Partial Knee' OR 'Unicompartmental Knee Arthroplasty' OR 'Arthroplasty, Unicompartmental Knee' OR 'Knee Arthroplasty, Unicompartmental' OR 'Unicondylar Knee Arthroplasty' OR 'Arthroplasty, Unicondylar Knee' OR 'Knee Arthroplasty, Unicondylar' OR 'Partial Knee Arthroplasty' OR 'Arthroplasty, Partial Knee' OR 'Knee Arthroplasty, Partial' OR 'Unicondylar Knee Replacement' OR 'Knee Replacement, Unicondylar' OR 'Partial Knee Replacement' OR 'Knee Replacement, Partial' OR 'Unicompartmental Knee Replacement' OR 'Knee Replacement, Unicompartmental'

Web of science：(((((((((((((((((((((((((((((((ALL=(Arthroplasty, Replacement, Knee)) OR ALL=(Arthroplasties, Replacement, Knee)) OR ALL=(Arthroplasty, Knee Replacement)) OR ALL=(Knee Replacement Arthroplasties)) OR ALL=(Knee Replacement Arthroplasty)) OR ALL=(Replacement Arthroplasties, Knee)) OR ALL=(Knee Arthroplasty, Total)) OR ALL=(Arthroplasty, Total Knee)) OR ALL=(Total Knee Arthroplasty)) OR ALL=(Replacement, Total Knee)) OR ALL=(Total Knee Replacement)) OR ALL=(Knee Replacement, Total)) OR ALL=(Knee Arthroplasty)) OR ALL=(Arthroplasty, Knee)) OR ALL=(Arthroplasties, Knee Replacement)) OR ALL=(Replacement Arthroplasty, Knee)) OR ALL=(Arthroplasty, Replacement, Partial Knee)) OR ALL=(Unicompartmental Knee Arthroplasty)) OR ALL=(Arthroplasty, Unicompartmental Knee)) OR ALL=(Knee Arthroplasty, Unicompartmental)) OR ALL=(Unicondylar Knee Arthroplasty)) OR ALL=(Arthroplasty, Unicondylar Knee)) OR ALL=(Knee Arthroplasty, Unicondylar)) OR ALL=(Partial Knee Arthroplasty)) OR ALL=(Arthroplasty, Partial Knee)) OR ALL=(Knee Arthroplasty, Partial)) OR ALL=(Unicondylar Knee Replacement)) OR ALL=(Knee Replacement, Unicondylar)) OR ALL=(Partial Knee Replacement)) OR ALL=(Knee Replacement, Partial)) OR ALL=(Unicompartmental Knee Replacement)) OR ALL=(Knee Replacement, Unicompartmental)

**Bacteriophage**

Pubmed：((("Bacteriophages"[Mesh]) OR (Bacteriophage)) OR (Phages)) OR (Phage)

Embase：'bacteriophage'/exp OR 'bacteriophage' OR 'Bacteriophage' OR 'Phages' OR 'Phage'

Web of science：(((ALL=(Bacteriophages)) OR ALL=(Bacteriophage)) OR ALL=(Phages)) OR ALL=(Phage)

*Other database searches refer to "pubmed".
